# Polymorphism of *MUC1* Gene in Vietnamese Gastric Cancer Patients: A Multicenter Case–Control Study

**DOI:** 10.3389/fonc.2021.694977

**Published:** 2021-08-31

**Authors:** Ngoc-Lan Thi Nguyen, Ngoc-Dzung Thi Dang, Quang-Huy Dang, Van-Chuc Tran, Hoang-Long Vo, Masamitsu Yamaguchi, Thanh-Van Ta

**Affiliations:** ^1^Biochemistry Department, Hanoi Medical University, Hanoi, Vietnam; ^2^Clinical Laboratory, Hanoi Medical University Hospital, Hanoi Medical University, Hanoi, Vietnam; ^3^Department of Medical Laboratory Science, Faculty of Medical Technology, Hanoi Medical University, Hanoi, Vietnam; ^4^Department of Scientific Research and International Cooperation, Hanoi Medical University Hospital, Hanoi Medical University, Hanoi, Vietnam; ^5^Department of Applied Biology, Advanced Insect Research Promotion Center, Kyoto Institute of Technology, Kyoto, Japan

**Keywords:** gastric cancer, polymorphism, rs4072037, rs2070803, *MUC1* gene, *Mucin 1* gene

## Abstract

**Background:**

A few studies revealed that the polymorphisms of *Mucin 1* gene have a role and significance as a susceptible factor contributing to gastric cancer. To better understand the roles of two *MUC1* genotype polymorphisms of rs4072037 and rs2070803 in the development of gastric cancer in Vietnamese population, a multicenter, large-sample, case–control study was conducted to investigate the potential association of these single-nucleotide polymorphisms (SNPs) of *MUC1* gene with gastric cancer risk and to evaluate the combination factors in relation with these SNPs.

**Methods:**

This case–control study included 302 gastric cancer patients and 304 controls at four national medical hospitals between 2016 and 2018. All participants were interviewed for sociodemographic characteristics, smoking and drinking status, and personal and family history of gastric diseases. Genotyping was done using polymerase chain reaction–restriction fragment length polymorphism analysis. The association of SNPs with gastric cancer was explored using logistic regression models.

**Results:**

AA genotype for rs4072037 was significantly associated with increased gastric cancer. Those with AA genotype had higher gastric cancer risk than had patients with AG (OR: 2.09, 95% CI: 1.48–2.96) and a combination of AG+GG (OR: 1.85, 95% CI: 1.33–2.56). In rs2070803, GG genotype increased gastric cancer risk when compared with AG (OR: 1.97, 95% CI: 1.39–2.80) and AG+AA (OR: 1.71, 95% CI: 1.23–2.39). AG genotypes in both SNPs decreased gastric cancer risk when compared with homogenous genotype, more specifically AA (OR: 0.51, 95% CI: 0.35–0.72) and GG (OR: 0.58, 95% CI: 0.35–0.97). These genotypes in combination with above-60-year-old age, male gender, alcoholism, and personal history of gastric disease were also significantly elevated risk factors for gastric cancer.

**Conclusions:**

rs4072037 and rs2070803 of *Mucin 1* genes are two genotypic risk factors for gastric cancer. Those in combination with gender, family history, smoking, and drinking habits significantly increase the risk of gastric cancer.

## Introduction

Gastric cancer, the fourth most common cancer worldwide and the second leading cause of cancer death, was known as a heterogeneous, multifactorial, highly malignant type of cancer (1). Whereas the burden of gastric cancer is no longer common in North America and in most Western European countries, it still remains great in Asia (1). Vietnam, a lower middle-income country in Southeast Asia with the shortcomings existing regarding the quality and resources of clinical practice in hospital-based conditions, was illustrated by the high incidence rate and poor prognosis of the disease that accounted for about 18,000 new cases and 15,000 deaths in 2018 (2). The multistep process of gastric carcinogenesis starts from chronic inflammation, followed by multifocal atrophic gastritis with intestinal metaplasia and dysplasia, and ends with gastric adenocarcinoma (3). Well-established pathological and environmental risk factors of gastric cancer include *Helicobacter pylori* (3, 4), smoking (5), and poor diet (6). In recent years, special focus has been shifted to the identification of genetic factors, characterized by single-nucleotide polymorphisms (SNPs), which exist in large numbers in the human genome (7–9). Several genome-wide association studies have indicated the association between gastric cancer and SNPs of *Mucin 1* (*MUC1*) gene, which codes for the cell surface glycoprotein mucin-1 and is used in clinical practice settings as tumor marker C15-3, in various Asian and European populations (7, 10, 11), besides two SNPs rs4072037 and rs2070803 of *MUC1* gene that were found to be associated with increased gastric cancer susceptibility. Despite a high incidence of gastric cancer in Vietnam, genetic association study with a particular interest in the association between gene SNPs and gastric cancer in Vietnamese population is, to date, still very sparse (12). Each population of gastric cancer in each country has its own unique environment- and lifestyle-related and individual characteristics. Gastric cancer, as with other neoplastic diseases, also follows a polygenic disease model, with multiple genes implicated across the populations. Different populations presented with different genetic compositions, which in turn meant different gene–gene interaction and gene–environment interaction. It is important that we replicate the study about *MUC1* polymorphism in Vietnamese population to understand with certainty whether it has different effects on the risk of developing gastric cancer and, in turn, developing a population-specific genetic panel for gastric cancer screening. It is, therefore, with great advances in our understanding of the genetic basis of gastric cancer, necessary to take advantage of the advancement in molecular targeted treatment technologies to gain an understanding of the genetic factors associated with the disease in order to develop new and more efficient therapeutic targets. To better understand the roles of two *MUC1* genotype polymorphisms of rs4072037 and rs2070803 in the development of gastric cancer in Vietnamese population, we sought to investigate the potential association of these SNPs of *MUC1* gene with gastric cancer risk and evaluate the combination factors in relation with these SNPs.

## Methods

### Study Design and Participants

This case–control study was conducted at four national medical hospitals (Hanoi Medical University Hospital, National Cancer Hospital, 108 Military Central Hospital, and Vietnam-Germany Hospital) in Hanoi, Vietnam. The study protocol was approved by the Ethics Council of Hanoi Medical University (decision number 198/HĐĐĐĐHYHN). This study was conducted in accordance with the Declaration of Helsinki. Written informed consent was obtained from all the study participants before they were interviewed. We included 302 cases and 304 controls from 2016 to 2018. The diagnosis of Vietnamese with gastric cancer was based on the histopathology confirmation for eligibility as cases. Regarding the cases in this study, we included newly diagnosed, untreated, and treated patients. The selected control group was composed of people endoscopically diagnosed with normal epithelial gastric or only acute gastritis. Our patients were selected independently from four institutions and were not blood relatives.

Each participant was scheduled for an interview with trained interviewers. Environmental information of study participants was collected, including gender, age, educational status, occupation, personal and family’s medical history, alcohol usage, and smoking habit. The variables of personal history were collected based on specific interview questions. Personal history of a disease was documented only when the patients have been diagnosed by the doctor. In particular, personal history of *H. pylori* was assessed based on i) asking about a history of whether they were diagnosed with *H. pylori* before, ii) information gathered from medical records, and iii) IgG serology test. The study patients were documented with the personal history of *H. pylori* if one of the three above results was positive, whereas the patients have no history of *H. pylori* infection if all three of the above results are negative. Family history of study participants included the family history of gastric cancer, the number of family members diagnosed with gastric cancer, their time of diagnosis, and their relationship with the participants. Smoking history and alcohol abuse history were investigated using the standards following the Centers for Disease Control and Prevention and the WHO, respectively (13, 14).

### DNA Extraction

Peripheral venous blood samples in EDTA containers were acquired from all subjects and were stored in appropriate condition before being transported to the Quality Control Center for Medical Laboratories, Hanoi Medical University, for gene analysis. We extracted the genomic DNA from peripheral blood lymphocytes using Exgene™ Blood SV Kit (GeneAll, Korea). Following the manufacturer’s protocol, we isolated the genomic DNA and stored it at −80°C until SNP analysis of *MUC1* gene.

Fifty percent of the samples were sent to the Kyoto Institute of Technology for gene analysis using the same method, and 10% of the samples were analyzed in both laboratories for cross-checking.

### Detection of *MUC1* Gene Polymorphism

The two *MUC1* genotype polymorphisms (rs4072037 and rs2070803) were determined by polymerase chain reaction–restriction fragment length polymorphism (PCR-RFLP) method with PCR primer pairs listed in **Table 1**.

**Table 1:** Specific primers (IDT, USA) according to *MUC1* polymorphisms.

rs4072037	5′-AAGGCCTATGGGCAGAGAGA-3′ (forward) 5′-ACGCTGCTGGTCATACTCAC-3′ (reverse)
rs2070803	5′-CTTAGCTGTCCGGGTGTGAAGT-3′ (forward) 5′-TGTGGTTCTAGGCAGGAGCAAC-3′ (reverse)

**Figure 1:**
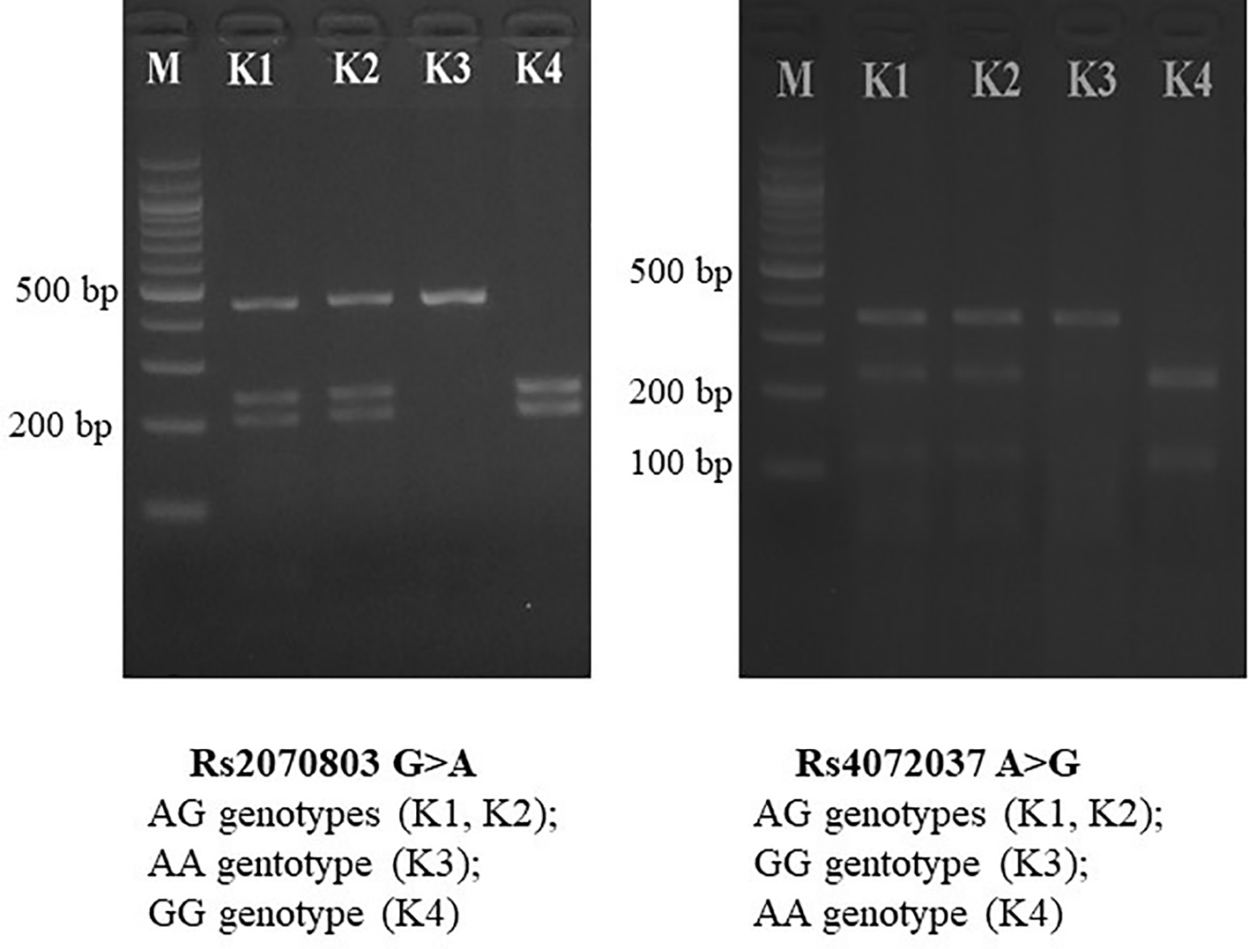
PCR-RFLP for MUC1 gene polymorphisms. PCR-RFLP, polymerase chain reaction–restriction fragment length polymorphism.

The PCR was carried out in a 30 μl reaction mix containing 100 ng of DNA template, 1 unit of Taq Mastermix (New England BioLabs, Beverly, MA, USA), 0.5 μM of forward primer, and 0.5 μM of reverse primer. The PCR was carried out in a thermocycler, and reaction conditions consisted of 95°C denaturation for 5 min, 94°C annealing for 30 s, 62°C annealing for 30 s, 72°C annealing for 30 s (40 cycles), and 72°C elongation for 10 min. The PCR products were digested with appropriate restriction enzymes (New England BioLabs, Beverly, MA, USA) at 37°C for 8 h. The restriction enzymes were *Alw*NI (rs4072037 G/A) and *Taq*aI (rs2070803 G/A). The PCR and digestion products were analyzed with a 1.5% agarose (Serva, Germany) gel with intercalating dye (ethidium bromide) staining. Selected PCR-amplified DNA samples (about 5%) were analyzed by DNA sequencing (**Figure 1**).

### Statistical Analysis

Data were analyzed using Stata 12.0 (StataCorp LP, College Station, TX, USA). All data first underwent a visual inspection for coding errors, outliers, or funky distributions. The frequency distribution of each variable was examined according to the case and control groups. Distribution of the two SNPs was performed following the Hardy–Weinberg equilibrium. Student’s t-test and chi-square were applied to evaluate the differences in the distributions of environmental variables, and genotypes of the rs4072037 and rs2070803 between the cases and the controls. Univariate and multivariate logistic regression models were employed to identify the associations between the three genotypes of SNPs and risk of gastric cancer. Odds ratios (ORs) with 95% confidence intervals (95% CI) were constructed. The significance level was set at 0.05.

## Results

### Environmental Characteristics of Participants

A total of 606 participants were analyzed in both groups, with males significantly outnumbering females by 2 to 1. There were significant differences between cases and controls in personal history of gastric cancer, family history of gastric cancer, personal history of *H. pylori*, and history of alcohol abuse (p < 0.05). In contrast, a high level of similarity in age and gender distribution was observed between two groups, with the p-values of 0.81 and 0.16 (**Table 2**).

**Table 2:** Frequency distribution of selected characteristics in gastric cancer cases and controls.

Variables	Control (n = 304)		Case (n = 302)		Total (n = 606)		p-Value
	n	%	n	%	n	%	
Gender							
Male	195	64.14	210	69.54	405	66.72	0.16
Female	109	35.86	92	30.46	201	33.28
Age							
<60	152	50.00	148	49.00	300	49.50	0.81
≥60	152	50.00	154	51.00	306	50.50
Personal history of gastric diseases							
No	111	36.51	140	47.30	251	41.83	0.00*
Yes	193	63.49	156	52.70	349	58.17
Family history of gastric cancer							
No	285	95.96	261	87.58	546	91.76	0.00*
Yes	12	4.04	37	12.42	49	8.24
History of alcohol abuse							
No	208	68.42	176	58.28	384	63.37	0.01*
Yes	96	31.58	126	41.72	222	36.63
Personal history of *Helicobacter pylori*							0.00*
No	123	40.46	191	63.25	314	51.82	
Yes	181	59.54	111	36.75	292	48.18	
Smoking							
No	179	58.88	159	52.65	338	55.78	0.12
Yes	125	41.12	143	47.35	268	44.22

*Significant at 0.05.

The logistic regression model of multiple environmental factors with gastric cancer is presented in **Table 3**. The family history of gastric cancer was significantly associated with increased gastric cancer risk (OR: 3.69, 95% CI: 1.78–7.66). Alcohol abuse (OR: 0.50, 95% CI: 0.26–0.95) and history of *H. pylori* infection (OR: 0.42, 95% CI: 0.29–0.61) were significantly inversely associated with gastric cancer. No significant associations for gastric cancer were observed in gender, age group, smoking, and personal history (p > 0.05).

**Table 3:** Association between environmental factors and risk of gastric cancer: multivariate logistic regression analysis.

	OR	95% CI	p-Value
Gender			
Female	Ref		
Male	1.56	0.92–2.64	0.098
Age group			
<60	Ref		
≥60	0.72	0.50–1.05	0.087
Smoking			
No	Ref		
Yes	1.05	0.66–1.65	0.842
Alcohol abuse			
No	Ref		
Yes	0.50	0.26–0.95	0.036*
History of *Helicobacter pylori* infection			
No	Ref		
Yes	0.42	0.29–0.61	0.000*
Personal history			
No	Ref		
Yes	1.42	0.99-2.04	0.056
Family history of gastric cancer			
No	Ref		
Yes	3.69	1.78–7.66	0.000*

Ref, reference group; OR, odds ratio; 95% CI, 95% confidence interval.

*Significant at 0.05.

### Distribution of Genes and Alleles

Significant variations in the distribution of genes and alleles were seen between cases and controls (p < 0.05). For rs4072037, the most common genotype in the case group was AA (49.34%), whereas the majority of patients were found to have AG genotype in the control group (53.29%). There was a significant difference in the frequency of alleles between two groups (p = 0.02). Similar to rs4072037, rs2070803 results indicated the differences in the genotypes between two groups, with the predominant identification of AG in the controls (53.95%) and GG in the cases (45.69%) (**Table 4**).

**Table 4:** Difference of the genotypes and allele distribution of rs4072037 and rs2070803 between gastric cancer cases and controls.

	Control (n = 304)		Case (n = 302)		p-Value
	n	%	n	%	
*rs4072037—MUC1*					
GG	37	12.17	43	14.24	0.00*
AG	162	53.29	110	36.42
AA	105	34.54	149	49.34
G allele	236	38.81	196	32.45	0.02*
A allele	372	61.18	408	67.55
*rs2070803—MUC1*					
AA	40	13.15	49	16.23	0.00*
AG	164	53.95	115	38.08
GG	100	32.89	138	45.69
A allele	244	40.13	213	35.26	0.09
G allele	364	59.87	391	64.74

*Significant at 0.05.

### Association of Genotypes and Alleles With the Risk of Gastric Cancer

In rs4072037, the individuals with AA genotype had higher gastric cancer risk than had patients with AG (OR: 2.09, 95% CI: 1.48–2.96) and a combination of AG+GG (OR: 1.85, 95% CI: 1.33–2.56). In rs2070803, GG genotype increased gastric cancer risk when compared with AG (OR: 1.97, 95% CI: 1.39–2.80) and AG+AA (OR: 1.71, 95% CI: 1.23–2.39). AG genotypes in both SNPs decreased gastric cancer risk when compared with homogenous genotype, more specifically AA (OR: 0.51, 95% CI: 0.35–0.72) and GG (OR: 0.58, 95% CI: 0.35–0.97) (**Table 5**).

**Table 5:** Association of the genotypes and alleles of rs4072037 and rs2070803 with the risk of gastric cancer: univariate logistic regression analysis.

	OR	95% CI	p-Value
*rs4072037*			
AG>GG	0.58	0.35–0.97	0.04*
AA>AG	2.09	1.48–2.96	0.00*
AA>AG+GG	1.85	1.33–2.56	0.00*
A > G	1.32	1.04–1.67	0.02*
*rs2070803*			
AG>AA	0.51	0.35–0.72	0.00*
GG>AG	1.97	1.39–2.80	0.00*
GG>AG+AA	1.71	1.23–2.39	0.00*
G>A	2.73	0.97–1.55	0.09

OR, odds ratio; 95% CI, 95% confidence interval.

*Significant at 0.05.

AA genotype of rs4072037 in combination with the factors including over the age of 60, male gender, alcohol abuse history, personal history gastritis, and family history of gastric cancer significantly elevated gastric cancer risk with adjusted OR from 1.57 to 6.47. AA genotype in combination with a family history of gastric cancer was the greatest risk factor for gastric cancer (OR: 6.47, 95% CI: 2.21–18.89). GG genotype of rs2070803 combined with family history of gastric cancer increased gastric cancer risk (OR: 6.18, 95% CI: 2.11–18.10) (**Table 6**).

**Table 6:** Association of the genotypes of two SNPs combined with environmental factors with the risk of gastric cancer: multivariate logistic regression analysis.

Risk Factors Combined With Genotypes	OR	95% CI	p-Value
*rs4072037*			
Age > 60 + AA	1.57	1.05–2.34	0.03*
Male + AA	1.84	1.28–2.67	0.00*
Smoking + AA	1.72	1.11–2.67	0.02*
Alcohol abuse history + AA	2.06	1.32–3.23	0.00*
Personal history of gastritis + AA	1.31	0.89–1.91	0.16
Family history of gastric cancer + AA	6.47	2.21–18.89	0.00*
*rs2070803*			
Age > 60 + GG	1.50	1.00–2.25	0.05*
Male + GG	1.80	1.25–2.63	0.00*
Smoking +GG	1.68	1.07–2.63	0.02*
Alcohol abuse history + GG	1.98	1.22–3.04	0.00*
Personal history of gastritis + GG	1.22	0.83–1.80	0.31
Family history of gastric cancer + GG	6.18	2.11–18.10	0.00*

OR, odds ratio; 95% CI, 95% confidence interval; SNP, single-nucleotide polymorphism.

*Significant at 0.05.

## Discussion

Two SNPs rs4072037 and rs2070803 of *MUC1* gene were found to be genotypic risk factors of gastric cancer. Those SNPs in combination with other environmental risk factors showed significantly increased risk for gastric cancer.

Genotypic distribution in the study population was found to be consistent with the results reported by Zhang and Jin and Song et al., both indicating AA being the most common genotype (15, 16). The present results revealed the association of allele A of rs4072037 with an increased risk of gastric cancer. Elevated gastric cancer risk was also found in the AA genotype group. Our findings were consistent with most previous reports by Xu et al. (17), Jia et al. (18), Palmer et al. (19), and Song et al. (15). Higher risk of cancer in patients with allele A compared with those with allele G was also pointed out by Saeki et al. (20). On the contrary, a decreased risk of gastric cancer in individuals with the G allele was shown in the study of Abnet et al. (21) and Shi et al. (22). Our result was similar to the reports from other Asian populations such as Japanese, Korean, and Chinese populations (OR ranging from 0.26 to 0.69) (16, 20, 23). In particular, in a recent meta-analysis with 10,092 gastric cancer cases and 15,236 controls, Peixi Liu clearly showed *MUC1* rs4072037 polymorphism was protective against the onset of gastric cancer (24). The rs4072037 located in the 5′ end of the second exon of *MUC1* gene allows determination of the splicing point. The G allele and the A allele belong to two different variants: two and three, respectively. *Via* the mutation of amino acids in the second exon, the structural differences between the two variants affect the N-terminal signal peptide, which, in turn, leads to a variation in the function of the encoded protein. The A allele associates with gastric cancer by lowering *MUC1* expression on the surface of epithelial cells lining the gastric mucosa. Mucins play a crucial role in forming protective mucous barriers on the epithelial surface of the stomach. The low expression of *MUC1* may increase the susceptibility to gastric cancer due to the reduced protective function of stomach (7, 20).

As for rs2070803, in comparison with the research of Li et al., despite a difference in the genetic distribution, there was a consistency regarding the higher risk of gastric cancer in GG genotype when compared with AA+AG genotype (25). The quantification of association between alleles and gastric cancer risk showed G compared with A with OR = 2.73 but no statistically significant association, while the study of Saeki et al. with three independent datasets (Tokyo, Aichi, and Korea) demonstrated a significant association between allele G and both intestinal and diffuse gastric cancer (20). This difference may be due to the limited sample size in our study. rs2070803 on the 1q22 chromosome is an SNP located between *MUC1* and *Tripartite Motif Containing 46* (*TRIM46*), both of which are located in a region of strong disequilibrium and are convergently transcribed (26). Numerous previous evidence showed the association between *MUC1* and the carcinogenesis of various tumor including gastric cancer (17, 18); however, there was no expression of *TRIM46* in the gastric mucosa. This suggests that rs2070803 is a tagging SNP for variants in *MUC1* gene, which is associated with gastric carcinogenesis.

Regarding the combinations of risk factors, old age (>60 years old) together with AA genotype of rs4072037 and GG genotype of rs2070803 showed a notable increase in gastric cancer risks. The majority of gastric cancer is primary and occurs in patients between the ages of 60 and 80, especially in Eastern Asia region (27, 28). Research on the Vietnamese population also reports a high incidence rate of gastric cancer in elderly people (12). The cancer growth process may involve several risk factors with different time and levels with which cells are affected. The affected cells need to be able to survive the apoptosis program in the immune system so that they can divide and multiply until tumors are formed. Due to the better immune system and shorter exposure time to most environmental risk factors that can accumulate gradually, young people tend to have a significantly lower risk of cancer than the elderly.

Gastric cancer risk was found to be increased roughly 1.8 times (p < 0.05) when both genotypic (rs4072037 AA genotype or rs2070803 GG genotype) and gender (male) factors were considered in our analysis. The gastric cancer incidence rate was reported to be approximately double in male compared with in female, particularly in the countries with a high prevalence of gastric cancer. Various characteristics such as smoking or alcohol drinking, which are attributed mostly to male patients, contribute to that fact (29). Another reason for the lower cancer rate in the female might be related to the hormone estrogen, which was reported to have a protective effect in decreasing stomach cancer risk (30–33). A multicenter cohort study in Korea pointed out similar results with isoflavone and phytoestrogen (34). This was further supported by several studies indicating an increased risk of gastric cancer in both genders treated with tamoxifen (an estrogen blocker) (35, 36). The result on the association between gastric cancer risk and a combination of *MUC1* polymorphisms (rs4072037 and rs2070803) and male gender once again showed that rs4072037 AA and rs2070803 GG were the two genotypic risk factor for stomach cancer. In our study, no notable association was found between history of alcohol abuse and gastric cancer; however, that factor together with rs4072037 AA genotype or rs2070803 GG genotype increased risk of gastric cancer significantly. The effect of alcohol on gastric cancer is still on debate. Several empirical studies suggested a carcinogenesis mechanism in which metabolic products of ethanol facilitate cancer risk factor penetration to damaged gastric mucosa, while other studies pointed out possible protective function of ethanol due to its destructive effect on *H. pylori* (36, 37). Research done on alcohol consumption rate and stomach cancer showed divergent results, with a few authors pointing to heavy drinking of various alcohol-based beverage, posing an even greater risk compared with standard alcohol (38–41).

Smoking, one of the primary risk factors, contributes to the manifestation of gastritis, atrophic gastritis, and gastric cancer in both the cardiac and non-cardiac regions. Nishino et al. reported 1.56 times higher risk in patients with a history of smoking (42). According to Gonzalez, approximately 18% of gastric cancer cases can be traced back to heavy smoking. In addition, gastric cancer risk has been found to increase with prolonged smoking time and decrease after 10 years of cessation (43). Tobacco smoke was proved to be a mixture of many harmful chemicals relating to human gastric carcinoma (44). The smoking-related DNA adducts that bind to DNA of the gastric mucous membrane have been found in samples from smokers (45). In our study, the smoking risk factor together with AA genotype of rs4072037 and GG genotype of the rs2070803 increased risk of gastric cancer. There are a number of studies supporting the high gastric cancer risk of patients who have a combination of genotypic risk factors (SNPs) and environmental factors (alcohol drinking/smoking). A report by Boccia et al. pointed out the increased gastric cancer risk in smokers with *Sulfotransferase Family 1A Member 1* (*SULT1A1*) gene and drinkers with CYRS2070803E1 gene (*5A allele or *6 allele) (46). *Cyclooxygenase-2* (*COX-2*) polymorphisms together with a history of smoking played an important role in the development of gastric cardia adenocarcinoma (47). TNF-alpha-857 C/T genotypic polymorphism was an independent risk factor, and gastric cancer caused by *tumor necrosis factor* (*TNF*) gene has been argued to be related to smoking habit (48). A study of Xu et al. demonstrated a *MUC1* mechanism, in which the inflammation signal was activated by macrophages, which contributed to the manifestation of lung cancer in smokers (49).

Family history of gastric cancer is known as one of the major factors that double or even triple the risk of gastric cancer (50, 51). The percentage of patients who had a family history of gastric cancer in our research was 12.4%, lower than the reports in an Italian population (21.9%) (52) and a Spanish population (17.6%) (53). A study of Dhillon (51) of 695 cases and 629 controls in America estimated the association between gastric cancer and family history with OR = 2.2 (95% CI: 1.5–3.3). This was further increased in individuals who have two or more family members diagnosed with gastric cancer, with OR = 12.1 (95% CI: 1.4–108) (51). Nine studies in the populations of Turkey, Italy, Finland, German, and Spain also showed significant association with OR ranging from 1.8 to 10.1, depending on different countries (54–57), besides five studies in a Japanese population that demonstrated the same association with OR from 1.5 to 3.5 (58, 59). Consistent result was also indicated in our study (**Table 3**), with OR = 3.69 (95% CI: 1.78–7.66). The detailed mechanism of the cause-and-effect relationship between family history and gastric cancer has not fully understood; however, special focus has been put on genetic characteristics. In our research, the individuals with a family history of gastric cancer in conjunction with rs4072037 AA genotype and rs2070803 genotype elevated gastric cancer risk significantly. This indicated the importance of family history of gastric cancer as a major risk factor for gastric cancer, especially in combination with other genotypic risk factors of rs4072037 and rs2070803. Therefore, a classification of patients according to different kinds of risk factor is necessary for the management, monitoring, and prevention of gastric cancer. This could be benefited from the implementation of a complete system focusing on the management of cancer among the individuals who have a history of cancer.

Several limitations should be acknowledged in this study. First, there was the lack of information on the *H. pylori* status of study participants. Second, the evaluation of histopathological characteristics of gastric cancer in different medical hospitals lacks uniform guidelines, which greatly affects the assessment of histopathological results. Third, we could not clarify genotypic risk patterns in combination with environmental risk factors due to incomplete data in our hospital cancer registry. Finally, later studies with larger sample size on the Vietnamese population are needed to confirm the effectiveness and accuracy of the model obtained from this study.

## Conclusions

In summary, this is the first large multicenter case–control study in Vietnamese population to investigate the effect of two SNPs rs4072037 and rs2070803 in *MUC1* gene as the risk factor for gastric cancer. We found the individuals carrying rs4072037 and rs2070803 polymorphisms would be more susceptible to gastric cancer. Importantly, those genetic factors in the interplay with some environmental factors such as smoking, alcohol abuse, and family history of gastric cancer significantly increased the risk of gastric cancer.

## Data Availability Statement

The raw data supporting the conclusions of this article will be made available by the authors, without undue reservation.

## Ethics Statement

The study protocol was approved by the Ethics Council of Hanoi Medical University (decision number 198/HĐĐĐĐHYHN). The patients/participants provided their written informed consent to participate in this study.

## Author Contributions

N-LN took part in designing the research, collected samples, analyzed and interpreted the participants’ data regarding SNPs of gastric cancer, and contributed to statistical analysis and writing the manuscript. N-DD took part in designing the research and writing the manuscript, with equal contribution with N-LN. Q-HD and H-LV were responsible for statistical analysis and writing the manuscript. V-CT collected samples, analyzed the samples, and wrote the manuscript. MY contributed to writing the manuscript and analyzed the samples. T-VT was responsible for research design and writing of the manuscript. All authors contributed to the article and approved the submitted version.

## Funding

This research is funded by the Vietnam National Foundation for Science and Technology Development (NAFOSTED) under grant number 106-YS.02-2015.37. The funding includes research design, samples, data collection, and gene analysis.

## Conflict of Interest

The authors declare that the research was conducted in the absence of any commercial or financial relationships that could be construed as a potential conflict of interest.

## Publisher’s Note

All claims expressed in this article are solely those of the authors and do not necessarily represent those of their affiliated organizations, or those of the publisher, the editors and the reviewers. Any product that may be evaluated in this article, or claim that may be made by its manufacturer, is not guaranteed or endorsed by the publisher.

## Abbreviations

*MUC1*, *Mucin 1*; SNP, single-nucleotide polymorphism; PCR-RFLP, polymerase chain reaction–restriction fragment length polymorphism; OR, odds ratio; *H. pylori*, *Helicobactor pylori*; *TRIM46*, *Tripartite Motif Containing 46*; *SULT1A1*, *Sulfotransferase Family 1A Member 1*; *COX-2*, *cyclooxygenase-2*; TNF, tumor necrosis factor.
